# Glycerylphytate compounds with tunable ion affinity and osteogenic properties

**DOI:** 10.1038/s41598-019-48015-5

**Published:** 2019-08-07

**Authors:** Ana Mora-Boza, María Luisa López-Donaire, Laura Saldaña, Nuria Vilaboa, Blanca Vázquez-Lasa, Julio San Román

**Affiliations:** 1 Institute of Polymer Science and Technology, ICTP-CSIC, C/Juan de la Cierva 3, 28006 Madrid, Spain; 2 CIBER-BBN, Health Institute Carlos III, C/Monforte de Lemos 3-5, Pabellón 11, 28029 Madrid, Spain; 3 Hospital Universitario La Paz-IdiPAZ, Paseo de La Castellana 261, 28046 Madrid, Spain

**Keywords:** Mesenchymal stem cells, Chemical biology

## Abstract

Phytic acid (PA) is a natural-occurring antioxidant, which plays an important role in many biological processes. PA is recognized as a potent inhibitor of lipid peroxidation because of its high affinity to multivalent cations, and it can play a role in osteogenic processes. However, its powerful chelating capacity is controversial because it can lead to a severe reduction of mineral availability in the organism. For this reason, compounds with beneficial biological properties of PA, but a modular ion binding capacity, are of high interest. In this work, we report the synthesis and physicochemical characterization of two hydroxylic derivatives of PA, named glycerylphytates (GPhy), through a condensation reaction of PA with glycerol (G). Both derivatives present antioxidant properties, measured by ferrozine/FeCl_2_ method and chelating activity with calcium ions depending on the content of glyceryl groups incorporated. Besides, the hydroxylic modification not only modulates the ion binding affinity of derivatives but also improves their cytocompatibility in human bone marrow mesenchymal cells (MSCs). Furthermore, GPhy derivatives display osteogenic properties, confirmed by *COL1A* and *ALPL* expression depending on composition. These positive features convert GPhy compounds into potent alternatives for those skeletal diseases treatments where PA is tentatively applied.

## Introduction

Phytic acid (PA), or myo-inositol hexakisphosphate, is a powerful natural-occurring antioxidant, which constitutes up to 85% of the total phosphorus content in legumes and most cereals^1,2^. PA is also present in mammalian cells as an endogenously synthesized compound that regulates essential biological processes^3–7^. As its name indicates, myo-inositol hexakisphosphate is a hexa-carbon carbohydrate with six phosphate groups, each one attached to a carbon group^8^. The presence of twelve ionizable protons in its structure makes PA form stable chelating complexes with multivalent cations conferring it a potent antioxidant power. Complexes are formed with transition metals but also with amine groups and proteins^1,9–12^. Iron-PA complexes formation has been widely studied since cell iron overload leads to the production of free radicals that can end in several degenerative processes^13^. Particularly, PA can maintain iron in the Fe(III) oxidation state by occupying all the available iron coordination sites and therefore, it can limit the formation of hydroxyl radicals^1^. Thus, PA has emerged as a powerful lipid peroxidation inhibitor^13–17^. This potent antioxidant character also makes PA play a role in diseases associated with bone loss due to the recently suggested connection between this type of pathogenesis and reactive oxygen species (ROS). ROS seem to promote osteoclastogenesis, and inhibit mineralization and osteogenesis processes^18^. In fact, an imbalance between the oxidant and antioxidant plasma biomarkers has been detected in patients with postmenopausal osteoporosis^18–20^. ROS are known to affect bone remodelling processes by activating NF-κB, a key factor involved in osteoclastogenesis^18^ which stimulates the expression of cytokines such as tumour necrosis factor-α (TNF-α) and interleukin-6 (IL-6)^18,21^. Therefore, taking into consideration the antioxidant properties of PA and the proposed influence of ROS in bone remodelling processes^18,21^, the use of PA as alternative treatment of musculoskeletal diseases is even of more interest.

On the other hand, bone mass loss is associated to an imbalance between new bone formation, mediated by osteoblasts, and bone resorption by osteoclasts^22,23^. In this respect, in recent years PA has arisen as an alternative antiosteoporotic agent as it has been claimed to play a key role in osteogenic processes^24^. Particularly, PA has demonstrated to be a potent *in vitro* inhibitor of osteoclastic activity, and it has also been suggested to modulate biomineralization^24,25^. However, its mechanism of action in osteoblasts activity remains unclear and could depend on the cell type^3,24,25^. Thus, Arriero *et al*. observed that PA treatment decreased the mineralization ability of mouse MC3T3-E1 osteoblastic cells without affecting the mRNA levels of genes involved in matrix maturation such as alkaline phosphatase (ALP), osteocalcin or bone sialoprotein. Adison *et al*. also observed that, at physiologic concentrations, PA causes a dose-dependent inhibition of mineralization in MC3T3-E1 cultures, which was related with increased mRNA levels of osteopontin, an inhibitor of mineralization, without affecting the expression of osteocalcin and bone sialoprotein^3^. Interestingly, treatment of human umbilical cord mesenchymal stem cells (hUC-MSCs) with PA increased *ALPL* expression under osteogenic conditions^24^.

Given the potential role of PA as a bone mass regulator, this paper is focused on the organic modification of PA by chemical anchorage of glyceryl moieties in different contents. This modification reduces the number of ionizable protons tuning the chelating activity with ions such as iron and calcium. It is expected that the novel chemical structure of hybrid phytate derivatives (named glycerylphytates, GPhy) also modulates some important biological properties such as cytocompatibility, cell-material interactions^26^, lipid oxidation and osteogenic activity. Thus, the synthesis of two novel hydroxylic PA derivatives was obtained by condensation reaction of glycerol (G) and PA in different feed molar ratios. GPhy products were characterized using Nuclear Magnetic Resonance (NMR), Attenuated Total Internal Reflectance Fourier Transform Infrared (ATR-FTIR), Energy Dispersive X-rays (EDX) and Induction Coupled Plasma (ICP) Spectroscopic techniques. The chelating activity of GPhy compounds to Fe^+2^ and Ca^+2^ ions was analysed. Finally, the *in vitro* biological effect of both glycerylphytate compounds was evaluated with human bone marrow mesenchymal cells (MSCs), measuring cytotoxicity, cell viability, ALP activity as well as the expression of key osteogenic genes.

## Results and Discussion

### Synthesis and characterization of GPhy derivatives

The main goal of the present work was to obtain phytate derivatives with improved cytocompatibility without sacrificing the interesting biological properties of PA, and at the same time, to enlarge their applications in the biomedical and pharmaceutical fields. Thus, two phytate derivatives have been prepared by introducing glyceryl moieties in the chemical structure of PA, obtaining two compounds that were named as G_1_Phy and G_3_Phy according to their G content, as it will be explained below. This type of strategy has been previously reported for different polyol-phosphate derivatives synthetized as prodrugs with enhancing bioavailability^26^.

**Figure 1:**
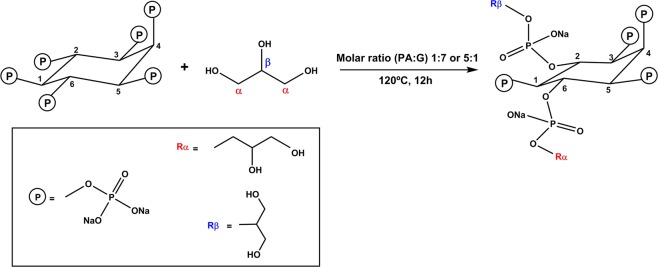
Synthetic procedure applied in the preparation of GPhy compounds showing a GPhy derivative in which the PA has reacted with two glycerol molecules through α and β hydroxylic groups, respectively.

Figure 1 displays the synthetic procedure applied in the preparation of GPhy. Based on the work of He *et al*.^27^ who proposed a 5-axial/1-equatorial structure of PA at alkaline solutions, we assumed a similar structure for solid PA. As illustrated in the synthetic scheme, there are different possibilities of reaction between the reactants due to the six phosphate groups in the PA structure and the two hydroxylic groups in the G molecule. In addition, other factors such as the PA:G ratio, the conformation of the cyclohexane ring and steric factors will have influence in the final reaction products^28^. In order to have an insight of the stoichiometry of the two condensates, structural characterization was performed. Table 1 shows the results obtained from Elemental analysis (EA) and ICP. The formulas were deduced taking also into consideration the results of loss of water and percentage of residue at 800 °C obtained from (Thermogravimetric Analysis) TGA of PA and both glycerylphytates. Results indicated an average number of 1 glyceryl residue for the compound obtained with a molar ratio PA:G of 5:1 and an average of 3 glyceryl groups for that obtained with a molar ratio PA:G of 1:7 (G_1_Phy and G_3_Phy compounds, respectively). This showed an agreement between the amount of G in the feed and the conjugation of glycerol moieties with phosphate groups. Additionally, the results suggested that the conjugation reaction was also determined by steric hindrance since the number of glyceryl conjugated moieties increased from 1 to 3 when the PA:G molar ratio varied from 5:1 to 1:7.

**Table 1:** Molecular empiric formula and elemental composition of GPhy derivatives.

Molecular formula^a^	C^b^		H^b^		Na^c^		P^c^	
	*Cal%*	*Found%*	*Cal%*	*Found%*	*Cal%*	*Found%*	*Cal%*	*Found%*
**Phytic Acid** C_6_H_12_O_24_P_6_·6Na·2H_2_O	8.70	8.34 ± 0.01	1.95	2.50 ± 0.02	16.66	15.26 ± 0.81	22.45	19.81 ± 1.59
**G**_**1**_ **Phy** C_9_H_18_O_26_P_6_·6Na·3 H_2_O	11.75	8.85 ± 0.03	2.63	2.99 ± 0.10	14.99	13.33 ± 0.72	20.20	17.10 ± 0.68
**G**_**3**_ **Phy** C_15_H_30_O_30_P_6_·6Na·2 H_2_O	17.16	17.36 ± 0.80	3.26	4.53 ± 0.10	13.13	10.34 ± 1.33	17.70	12.20 ± 1.84

^a^Determined by EA, ICP and TGA.

^b^Determined by EA.

^c^Determined by ICP spectroscopy.

EDX analysis confirmed the presence of expected elements and the absence of impurities (spectra not shown). The quantification of the elements content is shown in Fig. S1 and results mainly reflected an increase of carbon content in the G_3_Phy sample respect to those of PA and G_1_Phy as it was expected due to the higher G conjugation in the former derivative. These results correlated well with those obtained by EA.

**Figure 2:**
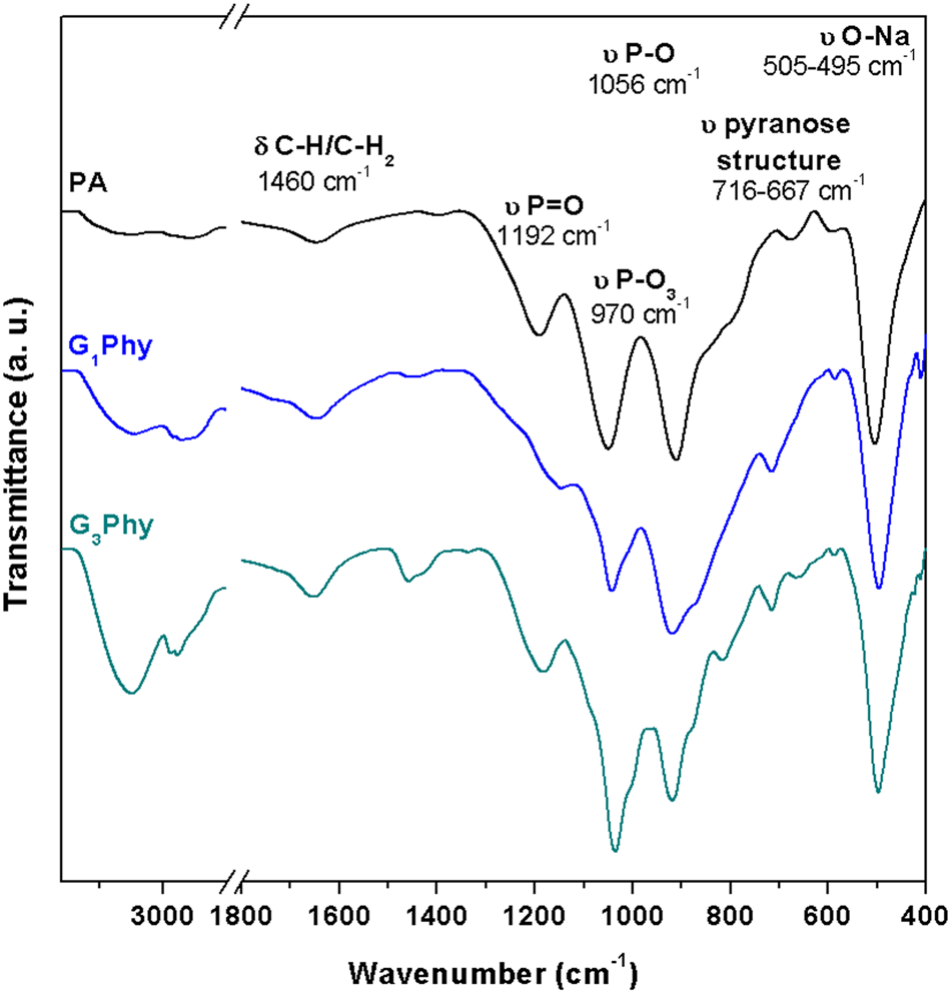
ATR-FTIR spectra obtained for PA, G_1_Phy and G_3_Phy.

Figure 2 shows the infrared spectra of PA and GPhy derivatives. PA spectrum showed a band at 3284 cm^−1^ due to the stretching vibrations of O–H groups, which suffered a broadening in the spectra of GPhy derivatives with the glyceryl content showing the contribution of the additional OH groups. The same observation was found in bands at 2974 and 2892 cm^−1^, corresponding to asymmetric and symmetric stretching vibrations of C–H bonds of inositol rings and glyceryl groups. The intensities of these bands increased in both GPhy compounds, but the rise was considerably remarkable in G_3_Phy due to the higher G conjugation. This feature was confirmed by the band at 1460 cm^−1^ attributed to δ CH_2_ groups in glyceryl groups as we can observe in the region of interest (1500–400 cm^−1^) in Fig. 2 ^29^. PA spectrum also showed a band at 1192 cm^−1^ corresponding to stretching vibration of P=O groups, and two bands at 1056 cm^−1^ and 913 cm^−1^, which were attributed to P–O and C–O–P stretching vibrations in COPO_3_ groups. Similar assignment for other phytates was given by Ishiguro *et al*.^30^ and Guan *et al*.^31^, who also assigned the two absorption bands at 1192 and 1056 cm^−1^ to asymmetric and symmetric stretching vibrations corresponding to P–O in HPO_3_^−^ groups in alkaline conditions. In the synthetized glycerylphytates these absorption bands shifted to lower wave numbers respect to PA. In particular, the band due to υ P=O groups shifted from 1192 cm^−1^ in the PA spectrum to 1185 cm^−1^ in the GPhy spectra, and the intense peak at 1056 cm^−1^ attributed to υ C–O alcohols, υ P–O and P–O–C, and υ C–O glycosidic, moved to 1035 cm^−1^ in the GPhy spectra. Finally, the absorption band at 970 cm^−1^ assigned to –PO_3_ groups, and peaks at 915 and 851 cm^−1^ assigned to υ P–O and P–O–C bonds shifted respect to PA. All these findings observed in the GPhy spectra indicated changes in the chemical environment of phosphate groups because of the condensation reaction^27,30,32^.

The GPhy compounds were subsequently characterized by NMR spectroscopy. The ^13^C NMR spectra of G_1_Phy and G_3_Phy and precursors are shown in Fig. S2a. The spectrum of PA showed four peaks at 74.7, 76.8, 77.5 and 78.9 ppm. The assignment of these peaks was performed according to previous work of Crimella *et al*.^33^ as follows: δ 75.3, C^4^ and C^6^; δ 78, C^2^; δ 78.5, C^1^ and C^3^; and δ 79.3, C^5^. The ^13^C-NMR spectrum of G showed a peak at 63.9 ppm due to CH_2_ carbons (C_α_) and a peak at 73.5 ppm assigned to the CH carbons (C_β_). Spectra of both derivatives were rather similar to each other and revealed notable changes in their signals respect to precursors, indicating the presence of different C atoms after the condensation reaction. ^13^C-NMR spectrum of G_3_Phy showed peaks at 63.6, 63.9, 67.3, 72.3, 72.6, 73.5, 73.7, 73.9 and 75.8 ppm. Signals in the range 63–68 ppm can be assigned to reacted C_α_ to form CH_2_–O–PO_3_– groups; the rest of the signals can be tentatively attributed to conjugated C_β_ in CH–O–PO_3_– groups of the derivative and CH–O–PO_3_– phytic carbons.

Figure S2b shows the ^1^H NMR spectra of both GPhy and precursors. Spectrum of G showed two doublet-of-doublet between 3.53 and 3.66 ppm that were assigned to no chemically equivalent H_α_ protons and a multiplet signal between 3.75 and 3.80 ppm assigned to H_β_ protons^34^. Spectrum of G_3_Phy showed modified signals in the range 3.50–3.82 ppm and two new multiplet signals at lower field; one of them was in the range 3.82–3.98 ppm and it was tentatively attributed to H_α_ and H_β_ protons in the groups CH_2_–O–P and CH–O–P of the glyceryl group. The other multiplet appeared in the range 4.05–4.10 ppm and it was attributed to H_β_ protons of CH–O–P groups in the glyceryl moieties. The spectra of G_1_Phy differed somewhat respect to that of G_3_Phy. The main difference lied in that the new multiplet in the range 3.82–3.98 ppm almost disappeared and the signal in the range 3.98–4.10 ppm attributed to resonance of H_β_ protons in glyceryl CH–O–P groups increased^33,35^. Proton NMR analysis of GPhy derivatives varying in glyceryl content suggested that for low G content in the feed, the reactivity of β hydroxylic groups towards phosphate groups would be favoured whereas an increase in the G content would enhance the reactivity of α hydroxylic.

**Figure 3:**
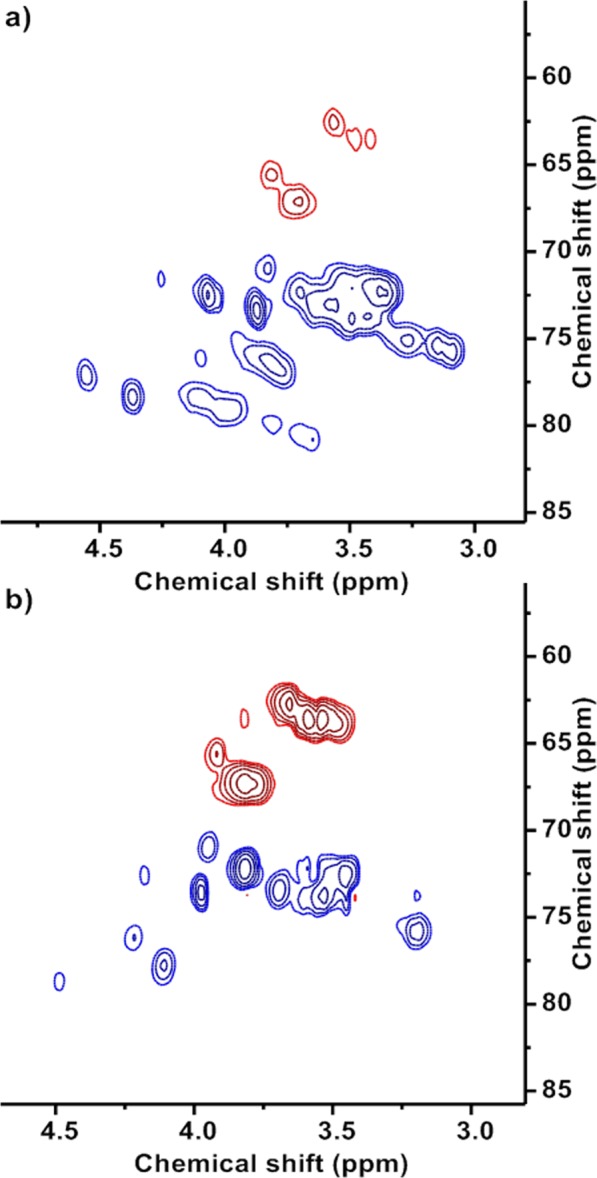
Two-dimensional HSQC spectra of ^13^C-decoupled DEPT of G_1_Phy (**a**) and G_3_Phy (**b**) recorded in D_2_O at 25 °C. For ^1^H experiments chemical shifts were referenced to the residual proton absorption of D_2_O (δ 4.79), while deuterated dioxane was used as external reference in ^13^C experiments. In ^13^C DEPT spectra, CH_2_signals are red while CH signals are blue.

This hypothesis was further studied through 2D DEPT-HSQC NMR analysis. In this concatenated experiment, the carbon multiplicity information obtained from the DEPT-experiment (CH or CH_2_ in our products) was detected in the 2D HSQC spectrum and illustrated in different colours (Fig. 3). In the DEPT spectra of both glycerylphytates, carbons in the range 60–67 ppm appeared in red colour (CH_2_) and carbons in the range 67–75 ppm appeared in blue colour (CH). Considering this information, in particular, in the 2D DEPT-HSQC spectrum of G_3_Phy the intense signal at 3.8/65.8 ppm was attributed to the glyceryl CH_2_–O groups conjugated with PA through CH_2_–O–P bonds and the signal at 3.8/70.7 ppm of lesser intensity, to conjugated CH–O groups of glyceryl moieties forming CH–O–P bonds. In addition, another two intense signals were present in the 2D spectrum, one of them at 3.5/62.2 ppm that could be due to CH_2_–O groups of reacted G, and the other one at 3.5/72.2 ppm due to reacted CH–O groups in the glyceryl moieties. As far as G_1_Phy is concerned, its 2D DEPT-HSQC spectrum showed that the majority of the signals were due to association of protons with CH carbons (blue colour). Moreover, the 2D plot of G_1_Phy showed less intense signals at 3.8/65.8 and 3.5/62.2 ppm as observed in G_3_Phy and instead, intense signals at 4.1/72.4 and 4.1/78.5 ppm. These findings suggest that conjugation reaction preferably proceeds between the β hydroxylic groups of G when this component is at low concentrations in the feed, whereas for higher G contents, the α hydroxylic groups are more likely to react, confirming the hypothesis previously proposed.

Thermal properties of GPhy derivatives were analysed by Differential Scanning Calorimetry (DSC) and TGA techniques. DSC thermograms revealed a glass transition temperature of 50 °C for both derivatives (thermograms not shown) indicating the amorphous character of these compounds because of the conjugation of glyceryl moieties in their structure. Table S1 summarizes the thermal degradation results of GPhy compounds and their precursors under inert atmosphere. Weight loss of glycerylphytates underwent in three stages in contrast to PA which showed four degradation steps. First stage was due to loss of water, 2 or 3 molecules for G_3_Phy and G_1_Phy, respectively, as explained above in the molecular formula analysis (Table 1). The second stage involved carbonization of conjugated glyceryl moieties and the dehydration of phytate structure by the decomposition of hydroxylic groups. Finally, the third and last degradation stage of GPhy (T_max_ around 340 °C) corresponded to further decomposition of the phytate moieties (332 °C)^36^. The residues of both derivatives obtained at 800 °C notably decreased in comparison to PA, indicating the content of glyceryl groups after the condensation reaction.

### Antioxidant properties

**Figure 4:**
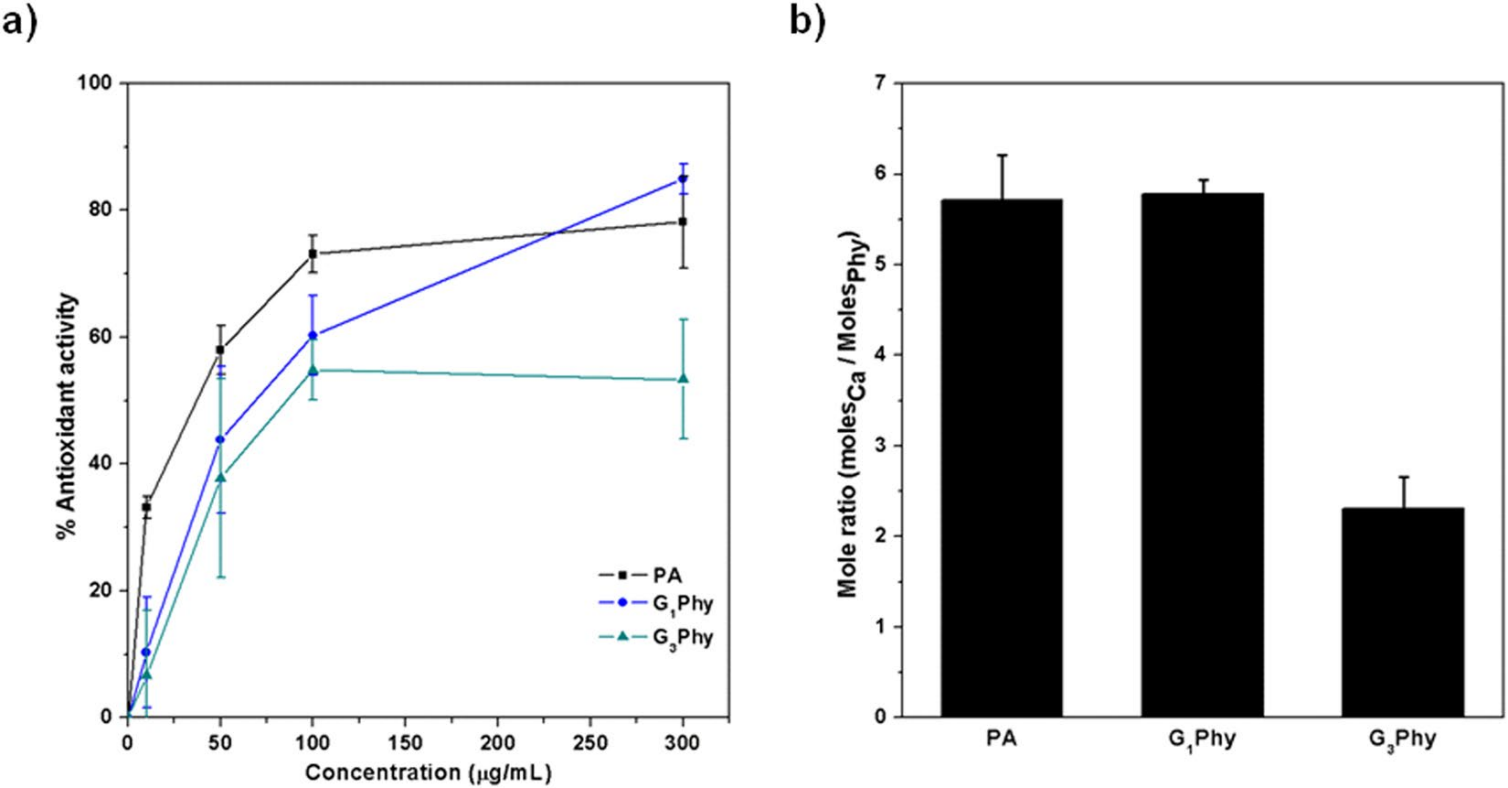
(**a**) Ferrous ions chelating activity effect of GPhy derivatives and PA at the indicated concentrations; (**b**) Chelating activity between Ca^2+^ and PA or GPhy compounds at pH 7.5.

PA exhibits a powerful antioxidant activity due to its ion chelating capacity and hence, it is able to prevent Fenton reaction by inhibiting iron-catalysed hydroxyl radical formation^1,14,16,37^. This property has repercussions on the bone mass loss, as it is known that iron-induced oxidative stress is directly linked to the activation of NF-kβ, a key element in osteoclastogenesis^18,21,38,39^. Then, to investigate the antioxidant properties of GPhy compounds, their chelating activity was examined by the ferrozine/FeCl_2_ system and compared with that of PA^15^. As it can be observed in Fig. 4a, PA and GPhy antioxidant properties were concentration dependent. Thus, ferrous chelating activity for PA was 33, 60, 73 and 78% at 10, 50, 100 and 300 µg/mL, respectively. Zajdel *et al*.^16^ reported similar results regarding the powerful inhibitory capacity of PA in iron-catalysed oxidative reactions. The GPhy compounds exhibited a significantly lower antioxidant activity than PA at the lowest tested concentration (10 µg/mL). However, their antioxidant activity increased with concentration in both compounds, achieving values of 60% and 54% for G_1_Phy and G_3_Phy respectively, at 100 μg/mL. In particular, the chelating activity of G_3_Phy at all tested concentrations was significantly (p < 0.01) lower compared to PA. G_1_Phy, on the other hand, exhibited similar behaviour to G_3_Phy in the range of 10–100 µg/mL, while antioxidant activity reached to 84% at 300 µg/mL, being no significantly different (p < 0.5) from PA value. These results indicate that antioxidant properties of GPhy compounds measured by ferrozine/FeCl_2_ method are not only concentration dependent as occurs with PA, but also composition dependent, showing antioxidant activity in the range of 50–80% for G_3_Phy and G_1_Phy respectively, at the highest studied concentration. This behaviour can be related to the incorporation of glyceryl moieties to PA structure, which reduces the number of ionizable protons and thus chelating properties.

### Calcium binding properties

Calcium deficiency plays a crucial role in bone-loss related diseases such as osteoporosis. Although calcium absorption can be affected with age, some dietary factors, and pharmaceutical compounds, can also act as potent chelating agents of calcium ions making them unavailable^40^. PA is shown to form insoluble complexes with Ca^2+^ ^7,41^, which can lead to a decrease in calcium uptake. In fact, some studies have been focused on the adverse effects of mineral uptake mediated by PA and its strong chelation capacity, concluding that the bioavaibility of these essential minerals can be compromised^40,42–44^. Particularly, magnesium and calcium deficiency has been linked to osteoporosis.

In this work, the Ca^2+^ binding capacity of GPhy derivatives was studied and compared with that of PA. After dropping a concentrated solution of the tested compound into a 4 mM solution of CaCl_2_, the precipitation of a solid, white or brown for PA and GPhy compounds respectively, was observed. The pellets were isolated and analysed by ICP spectroscopy. In Fig. 4b, the stoichiometry of the Ca-phytate complexes at pH 7.5 is expressed in moles_Ca_/moles_Phy_ where Phy can be PA, G_1_Phy or G_3_Phy. The stoichiometry of Ca-G_1_Phy complexes was quite similar to that of Ca-PA complexes. Interestingly, a notable decrease in the chelating capacity of G_3_Phy was obtained. This can be explained by the higher content of glyceryl moieties in this derivative in comparison to G_1_Phy. These results confirm that due to the introduction of glyceryl groups, G_3_Phy derivatives show lower chelating activity than PA and they show how this modification can tune their binding ability.

### *In vitro* biological effect of GPhy derivatives

#### Lipid peroxidation assay on RAW264.7 cells

**Figure 5:**
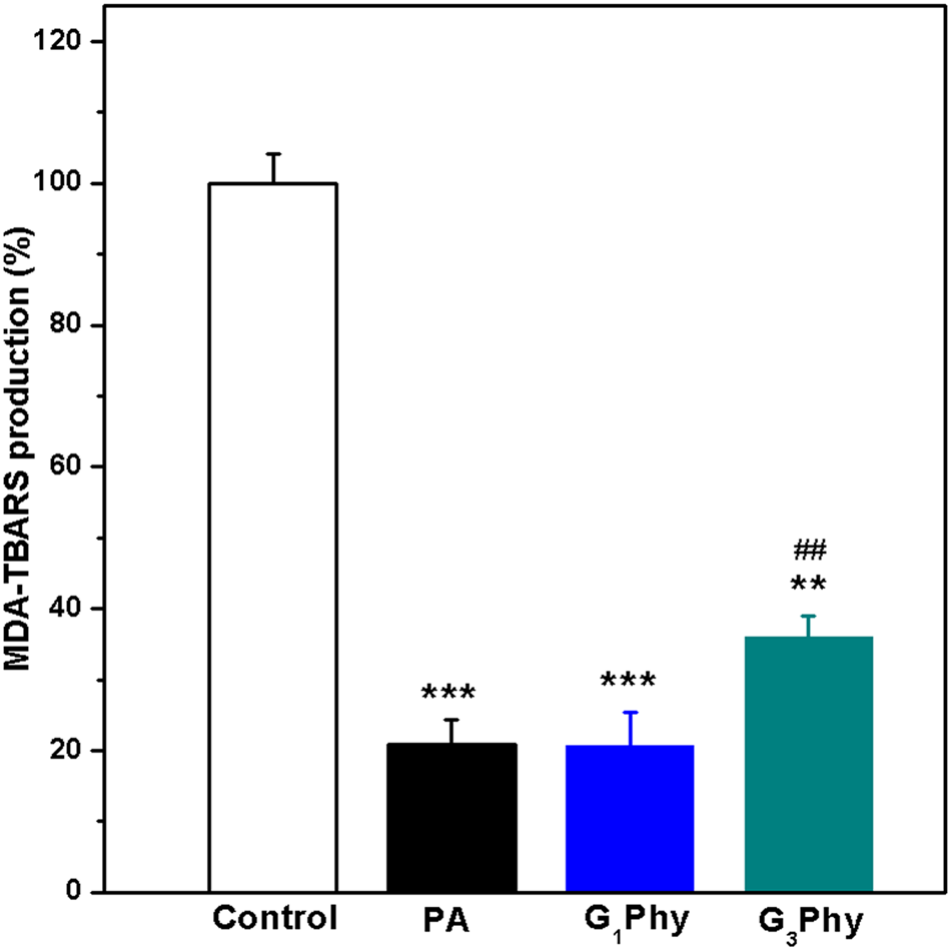
Effect of PA and its derivatives on MDA production. RAW267.4 cells were incubated overnight in presence of PA, G_1_Phy and G_3_Phy at a concentration of 100 μg/mL. Lipid peroxidation was induced with Fe^2+^/H_2_O_2_ system. Analysis of variance (ANOVA) of the results for tested samples was performed with respect to positive control at significance levels of **p < 0.01 and ***p < 0.001, and respect to PA at significance level of ^##^p < 0.05.

Lipid peroxidation inhibition was measured *in vitro* by MDA-TBA adduct formation after the induction of Fenton reaction with the Fe^2+^/H_2_O_2_ system on RAW264.7 cells. Figure 5 shows the results of the inhibitory effect on lipid peroxidation for PA and its derivatives at 100 μg/mL concentration. A higher amount of MDA-TBA adduct is indicative of a higher oxidative stress, therefore a 100% MDA-TBA was the maximum lipid peroxidation induced in the cells (positive control). When cells were incubated with PA or its derivatives, iron-loaded related stress was reduced due to their strong ability to chelate iron species. A higher MDA concentration was observed for G_3_Phy as compared to PA, which indicated a decrease in the ability to chelate iron ions as consequence of the higher content of glyceryl groups in its structure. Nevertheless, this compound was still able to prevent lipid peroxidation, as previously described for hydrolysis products of PA which maintain three or more phosphate groups^45,46^. Accordingly, G_3_Phy exhibited reasonable antioxidant iron stress properties in RAW264.7 cells in comparison to positive control. On the other side, G_1_Phy showed lipid peroxidation inhibition results comparable to PA, which correlated well with its antioxidant data obtained in previous analysis (Fig. 5). These results demonstrate that G_1_Phy and G_3_Phy maintain and modulate both iron (II) chelation and antioxidant properties of PA in cell cultures using the RAW264.7 cell line.

#### Cytotoxicity on MSCs

Biological properties of PA have been widely studied in different *in vitro* and *in vivo* models^2,47–50^, Using healthy cells, Norhaizan *et al*.^49^ reported that PA extracted from rice bran was not toxic up to 6 mM for 72 h in untransformed cultures of 3T3 cells, yielding < 10% of dead cells^49^, Similar results were reported by de Lima *et al*.^50^ who did not observe PA cytotoxicity at 4.0 mM for 48 h in lymphocytes isolated from healthy human donors. In this work, cytotoxicity (IC_50_) of PA and derivatives was tested in MSCs cultures using a standardized Alamar Blue assay. Viability of cells treated with G_1_Phy or G_3_Phy increased respect to PA. IC_50_ value of PA was 12.53 ± 2.21 mg/mL (14.5 ± 2.55 mM), while IC_50_ values of G_1_Phy and G_3_Phy were 22.81 ± 2.64 (23.41 ± 2.7 mM) and 29.81 ± 2.43 mg/mL (27.45 ± 2.23 mM), respectively. These results suggest that larger amounts of glycerylphytate derivatives than of PA could be safely used in different specific biomedical applications.

#### Osteogenic properties and biomineralization on MSCs

**Figure 6:**
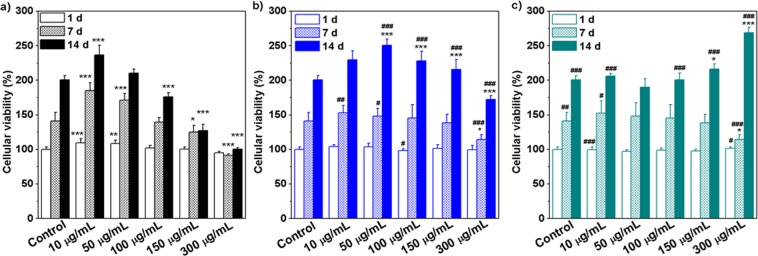
Cell viability (%) of PA (**a**), G_1_Phy (**b**) and G_3_Phy (**c**) at different concentrations for 1, 7 and 14 d. Mean ± SD values are relative to untreated MSCs culture for 1 d, which are given an arbitrary value of 100. Analysis of variance (ANOVA) of the results for tested samples was performed with respect to control (untreated cells) at each concentration and time at significance levels of *p < 0.05, **p < 0.01 and ***p < 0.001, and respect to PA at each concentration and time at significance levels of ^#^p < 0.05, ^##^p < 0.01 and ^###^p < 0.001.

The osteogenic activity of glycerylphytates and PA was investigated in MSCs cultures using Alamar Blue assay. To that end, firstly MSCs cultures were treated with the tested compound at different concentratios for 1, 7 and 14 d. Figure 6 shows that the metabolic activity of treated cells was affected by each tested compound in a different trend depending on dose and time. Cellular viability progressively decreased with concentration and time at 7 and 14 d and the lowest values were obtained at 150–300 µg/mL. G_3_Phy did not affect cell viability and even enhanced it at 300 µg/mL after long-term incubation time (14 d). The explanation of this enhancement is not clear yet but could be related with the less cytotoxicity of G_3_Phy which has an IC_50_ value nearly double than that of PA. The behaviour of G_1_Phy approached that of G_3_Phy for a concentration range between 10 and 150 µg/mL and only a deleterious effect was observed when cells were treated for 14 d with the highest tested dose. These findings indicate that composition of the developed glycerylphytates plays a dominant role in their *in vitro* cytocompatibility and support previous results on their less cytotoxicity respect to PA.

**Figure 7:**
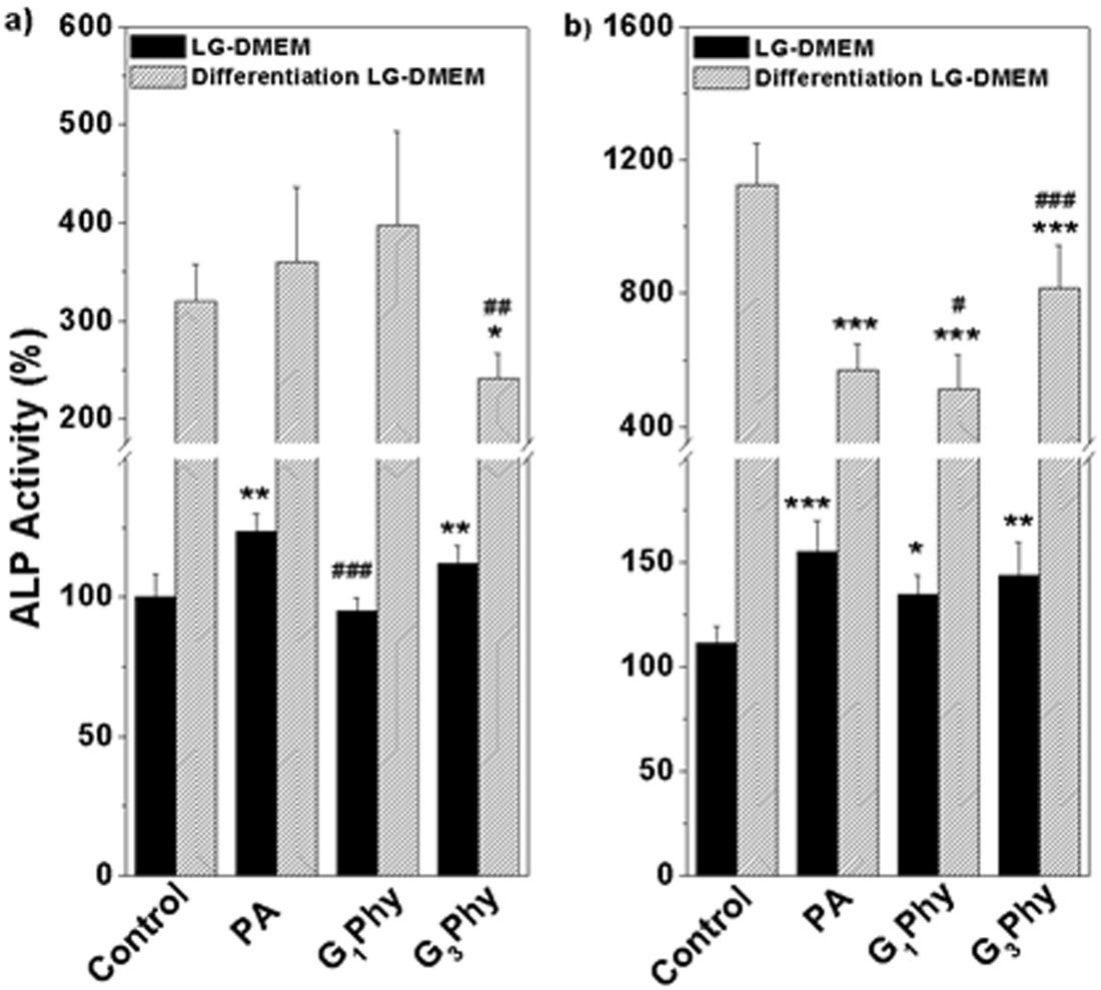
ALP activity normalized respect to DNA amount of MSCs treated for 7d (left) or 14 d (right) with 10 μg/mL of PA, G_1_Phy or G_3_Phy. MSCs were incubated with PA or its derivatives in complete LG-DMEM (solid bars) or differentiation LG-DMEM (striped bars). Values represent the mean ± SD. Analysis of variance (ANOVA) of the results for tested samples was performed with respect to the corresponding control at each time point and condition at significance levels of *p < 0.05, **p < 0.01 and ***p < 0.001 and with respect to PA samples at each time and condition at significance levels of ^#^p < 0.05, ^##^p < 0.01 and ^###^p < 0.001.

Since PA has been suggested to be involved in osteogenesis^3,5^, ALP activity, an early marker of osteoblast differentiation, was determined in MSCs treated with the compounds. Figure 7 depicts ALP activity of MSCs treated with 10 μg/mL PA or glycerylphytate compounds for 7 and 14 d in complete or differentiation LG-DMEM media. At the two incubation periods, ALP activity was higher in MSCs cultured in differentiation LG-DMEM than in complete LG-DMEM, either they were untreated or treated with compounds. Interestingly, a different response to the compounds was observed depending on the type of medium. In complete LG-DMEM, ALP activity of cells treated with PA or its derivatives slightly increased respect to control, except for the case of G_1_Phy, in which ALP remained unaffected after 7 d. However, a different behaviour was observed in MSCs cultured in differentiating medium. ALP activity of cells treated with PA or G_1_Phy for 7 d was similar to control, while treatment for 14 d resulted in lower ALP activity. Cells incubated with G_3_Phy showed an interesting behaviour as ALP activity was slightly lower at 7 d respect to control and PA, but at 14 d it reached higher values than those found for PA and G_1_Phy.

The mechanism by which PA participates in osteogenic processes is not well understood, but we can elucidate from these results that bioavailability of phosphate residues may be quite relevant. Furthermore, Addison *et al*.^3^ proposed that the high negative charge of PA molecule also has an impact on cellular interaction and diffusion across the membrane. For this reason, a possible explanation can lie in the enhanced cell-material interactions for G_3_Phy compound due to its major organic content. This hypothesis can be supported by the fact that this kind of modifications has been traditionally used for the protection of active phosphate groups, as those from PA^26^. An improved cytocompatibility would also enhance the metabolic effects that PA can trigger, as it has been demonstrated in this work. Thus, we can conclude that composition of GPhy compounds can tune the differentiation process of MSCs in comparison to PA itself.

#### Gene expression of osteogenic markers by RT-PCR

**Figure 8:**
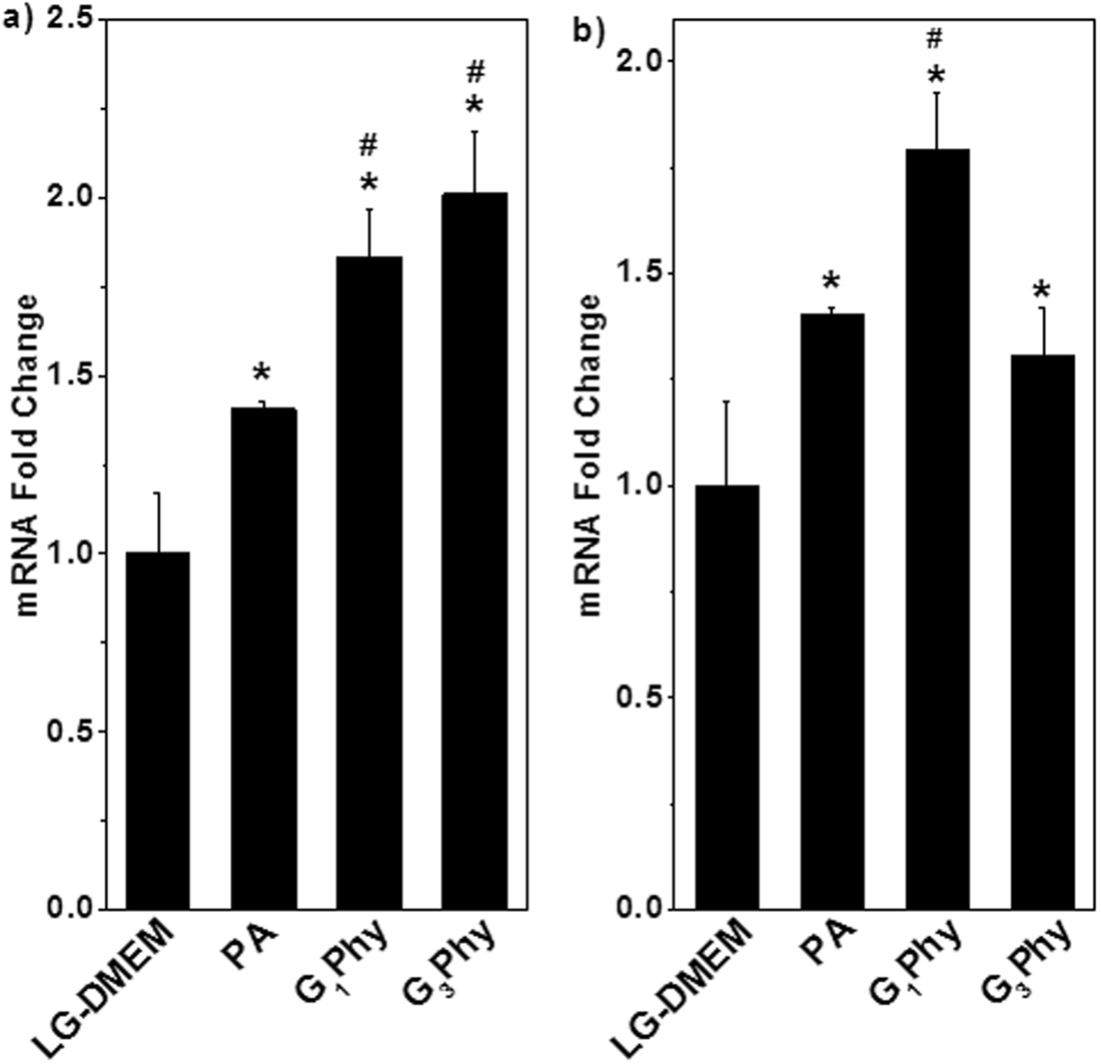
*COLA1A* (**a**) and *ALPL* (**b**) relative mRNA levels were determined after treating MSCs for 14 d with 10 μg/mL of PA, G_1_Phy or G_3_Phy. MSCs were incubated with the tested sample in complete LG-DMEM. Mean + SD values are relative to untreated MSCs, which were given an arbitrary value of 1. Analysis of variance (ANOVA) of the results for tested samples was performed with respect to untreated cells at significance levels of *p < 0.05, and with respect to PA treated cells at significance levels of ^#^p < 0.05.

As previously mentioned, treatment of MSCs with PA or GPhy for 14 in complete LG-DMEM d enhanced ALP activity (Fig. 7b). Thus, further studies investigated whether these compounds regulate the expression, at the mRNA levels, of osteogenic differentiation markers in MSCs. Results showed that incubation of MSCs with PA for 14 d in complete LG-DMEM increased *COL1A* expression (Fig. 8a). Interestingly, *COL1A* mRNA levels in MSCs treated with G_1_Phy or G_3_Phy were higher respect to PA suggesting that GPhy compounds could potentiate the deposition of a collagen matrix, which nucleates mineral deposition during osteogenesis. On the other side, a previous study reported that treatment of hUC-MSCs with 4 µM PA for 14 d under osteogenic conditions increased *ALPL* expression^24^. Data obtained in the present work indicated that incubation of MSCs with higher doses (10 μm) of PA in complete LG-DMEM induced an increase in *ALPL* mRNA levels, suggesting that PA could promote the acquisition of osteoblastic features in MSCs populations even in the absence of osteogenic inductors. Notably, G_1_Phy and G_3_Phy also increased *ALPL* expression in MSCs and *ALPL* transcript levels in MSCs treated with G_1_Phy were higher than those obtained in G_3_Phy or PA treated cells (Fig. 8b). It was not surprising that higher *ALPL* mRNA levels in G_1_Phy treated MSCs were not associated with increased ALP activity levels (Fig. 7b), as gene expression at the transcriptional levels does not necessarily correlate with protein activity, which is regulated by multiple post-transcriptional and post-translational mechanisms. Taken together, our results showed that PA and its derivatives might enhance MSC differentiation toward the osteoblastic lineage by regulating the expression of genes involved in matrix formation and maturation.

## Conclusions

In this paper, we report two new glycerylphytate derivatives (G_1_Phy, and G_3_Phy) which have different content of glyceryl groups. The reaction conditions allow to modulate chelation properties of the compound through the amount of glyceryl moieties incorporated. The new derivatives have improved cytocompatibility and osteogenic properties in comparison to PA in MSCs. Besides, both of them possess antioxidant and *in vitro* lipid peroxidation inhibition properties, which are essential features in tissue remodelling processes.

In summary, the chelation activity and biological properties of glycerylphytate compounds can be tuned by the organic content. This approach can offer a library of compounds with different composition to be used as an alternative to PA in biomedical and pharmaceutical applications related with stress oxidative phenomenon and low bone mass density.

## Methods

### Synthesis of GPhy derivatives

PA sodium salt hydrate (PA) and glycerol (G) were purchased from Sigma-Aldrich and used without further purification. The synthesis of GPhy compounds was carried out at 120 °C in bulk by reacting PA with the corresponding G volume for 12 h. The reaction products were dissolved in water, precipitated twice in 2-propanol, dried under reduced pressure to remove 2-propanol trace and lyophilized. Two GPhy derivatives were prepared using PA:G molar ratios of 5:1 and 1:7 which were named as G_1_Phy and G_3_Phy respectively.

### Physic-chemical characterization techniques

NMR experiments were performed using a Bruker AVANCE IIIHD-400 (399.86 MHz) spectrometer. Proton (^1^H-NMR) and Carbon 13 (^13^C-NMR) spectra were recorded in deuterated water (D_2_O) at 25 °C. ^1^H chemical shifts were referenced to the residual proton absorption of the solvent listed as “residual internal D_2_O (δ 4.79)”. The acquisition conditions were as follows: spectral windows, 20.04 ppm; pulse width, 14 µs; and 128 scans with a recycle delay of 1 s. In case of ^13^C, deuterated dioxane was use as external reference. The acquisition conditions were as follows: spectral windows, 396 ppm; pulse width, 15 µs; and 20000 scans with a recycle delay of 2 s between acquisitions. Two dimensional (2D) Heteronuclear Single-Quantum Coherence (HSQC) were recorded performing Distortionless Enhancement Polarization Transfer (DEPT) experiments (2D DEPT-HSQC). In this case, the acquisition conditions were: sweep width 165.00 ppm and 15.99 ppm for F_1_ and F_2_ dimensions, respectively; 256 and 8 scans for F_1_ and F_2_ dimensions, respectively. All measurements were conducted at 25 °C.

ATR-FTIR spectra were obtained on a Perkin-Elmer (Spectrum One) spectrometer equipped with an ATR accessory for all samples. EA was performed with an elemental LECO model CHNS-932 microanalyzer. EDX spectrometry analysis was performed with a Bruker XFlash model with detector 5030. Inductively coupled plasma optical emission spectrometry (ICP-OES) measurements were carried out in a 4300 DV Perkin-Elmer plasma emission spectrometer using a Gemcone (Perkin-Elmer) nebulizer. DSC experiments were carried out on a DSC model 8500 (PerkinElmer). Three heating-cooling cycles were analysed between 25 °C and 180 °C with a scanning rate of 10 °C/min under nitrogen at 20 mL/min flow rate. From the thermograms, the glass transition temperature (T_g_) was determined as the midpoint of the transition. TGA was performed in a thermogravimetric analyzer TGA Q500 (TA instruments) apparatus, under dynamic nitrogen at a heating rate of 10 °C/min in a range of 40–800 °C.

### Formation of ions chelate complexes

The iron chelate complexes formation was studied by the method of Dinis *et al*.^15^ PA and GPhy compounds at various concentrations (10–300 µg/ml) in dH_2_O (0.4 mL) were added to a solution of 2 mM FeCl_2_ (0.05 mL, Sigma Aldrich). The mixture was shaken vigorously and left at room temperature for 15 min under darkness conditions. Then, 0.2 mL of 5 mM ferrozine (Sigma Aldrich) solution was added, and the total volume was adjusted to 4 mL with ethanol. Absorbance of the solution was measured spectrophotometrically at 562 nm with a NanoDrop™ spectrophotometer. 10 mM EDTA solution and dH_2_O were used as positive and negative controls, respectively. The experiments were conducted in triplicate for each sample and the data obtained were expressed as mean values ± standard deviations (SD).

The binding capacity of PA and GPhy derivatives to calcium ions was examined with an adapted method from Saw *et al*.^51^. Particularly, aliquots of 50 µl at a concentration of 396 mg/mL of each compound were added to a 20 mL of a 4 mM solution of CaCl_2_ in intervals of 2 min until achieving a final concentration of 5.27 mg/mL of each compound in the solution. The pH was controlled by preparing all the solutions in Tris-HCl 0.1 M buffer pH 7.5. The pellet was isolated by centrifuging at 4 °C and 8000 rpm (Eppendorf centrifuge 5810 R model) for 10 min, and washed with dH_2_O. The final pellet was digested at 65 °C with 65% v/v HNO_3_. Finally, the phosphorus and calcium content of these pellets in 5% v/v HNO_3_ solution were measured by ICP. The experiments were conducted in triplicate for each sample and the data obtained were expressed as mean values ± SD.

### Cell culture

Human mesenchymal stem cells from bone marrow (MSCs, Innoprot) were grown and maintained in Mesenchymal Stem Cell Medium Kit (Innoprot) at 37 °C in a humidified atmosphere of 5% CO_2_. Cell culture media was refreshed every 48 h. For subsequent experiments, MSCs were cultured in Low Glucose Dulbecco’s Modified Eagle Medium (LG-DMEM), supplemented with 20% fetal bovine serum (FBS), 200 mM L-glutamine, 100 units/mL penicillin and 100 µg/mL streptomycin (complete LG-DMEM). Differentiation medium consisted of complete LG-DMEM supplemented with dexamethasone (100 nM), ascorbic acid (50 μg/mL), and β-glycerolphosphate (10 mM). All the experiments were performed using MSCs at passages 4 to 8.

Murine RAW 264.7 macrophage cell line (Innoprot) were grown and maintained in DMEM supplemented with 10% FBS, 200 mM L-glutamine, 100 units/mL penicillin and 100 µg/mL streptomycin, at 37 °C in a humidified atmosphere of 5% CO_2_. Cell culture media was refreshed every 48 h and these cells were used at passages 5 to 6 for all the experiments.

### Lipid peroxidation assay

Lipid peroxidation inhibition was tested in RAW264.7 cells, as macrophages are the major sources of oxidative stress and are used to evaluate the antioxidant function of dietary natural compounds^52^. 2 × 10^6^ RAW264.7 cells were seeded in 6-well plates and incubated for 24 h with the corresponding solution of PA, G_1_Phy or G_3_Phy at final concentration of 100 μg/mL. For positive (lipid peroxidation induction but without any studied agent) and negative (no lipid peroxidation induction) controls, the same volume of dH_2_O than for the studied samples was added. Lipid peroxidation was induced by incubating the samples with 50 μM Fe_2_SO_4_·7H_2_O and 200 μM H_2_O_2_ solution at 37 °C for 4 h in order to trigger the Fenton reaction^53^. Lipid peroxidation was evaluated using the Lipid Peroxidation Assay Kit (abcam), following the manufacturer instructions. The percentage (%) of lipid peroxidation inhibition was calculated respect to the positive control, considering it as the 100% of MDA producer. The experiments were conducted in triplicate for each sample and the data obtained were expressed as mean values ± SD.

### Cytotoxicity

Cytotoxicity of PA and GPhy compounds was evaluated in MSCs cultures using the Alamar Blue assay. A stock solution of the corresponding tested compound in DMEM lacking FBS and containing 2% w/v D-sorbitol was prepared and successive dilutions were tested (5, 15, 20, 30, 50 mg/mL). 9000 of MSCs were seeded in 96-well plates and incubated for 24 h. Then, the medium was replaced and the cells were treated with the tested compounds for 24 h. A 10% v/v Alamar Blue (Invitrogen) solution was prepared in DMEM without phenol red and the plates were incubated at 37 °C for 3 h. Medium was collected and, after laser excitation at 590 nm, emitted fluorescence at 530 nm was quantified using a Biotek Synergy HT plate reader (Biotek Synergy HT spectrophotometer).

### Cell viability

Alamar Blue assay was also used to analyse cell viability after incubation of MSCs with the tested compounds for 3, 7 and 14 d of. For this experiment, 104 of MSCs were seeded in 48-well plates and cultured in Mesenchymal Stem Cell Medium Kit for 24 h. Then, the medium was replaced, and the cells were treated with the tested solutions at various concentrations in complete LG-DMEM (10, 50,100, 150, and 300 µg/mL), and media containing the corresponding compounds were refreshed every 2 d. Medium was collected and, after laser excitation at 590 nm, emitted fluorescence at 530 nm was quantified using a Biotek Synergy HT plate reader (Biotek Synergy HT spectrophotometer). The experiments were conducted in triplicate for each sample and the data obtained were expressed as mean values ± SD.

### ALP activity quantification

ALP activity was evaluated after 3, 7 and 14 d of incubation of MSCs with the tested compounds. Total DNA amount was measured using the PicoGreen dSDNA quantitation kit (Molecular Probes). Determination of the ALP/DNA ratio is indicative of the amount of ALP activity per cell. For this experiment, 10^4^ of MSCs were seeded in 48-well plates and cultured in Mesenchymal Stem Cell Medium Kit for 24 h. Then, the medium was replaced, and the cells were treated with PA and its derivatives solutions at 10 µg/mL. The media containing the corresponding compounds were refreshed every 2 d. Negative and positive controls correspond to cells cultured in complete or differentiation LG-DMEM media, respectively. The experiments were conducted in triplicate for each sample and the data obtained were expressed as mean values ± SD.

### Analysis of differential gene expression by reverse transcription (RT) and real-time quantitative PCR (qPCR)

For this experiment, 10^6^ of MSCs were seeded in 6-well plates and cultured in complete Mesenchymal Stem Cell Medium Kit for 24 h. Then, the medium was replaced, and the cells were treated with PA and its derivatives solutions at 10 µg/mL in complete LG-DMEM. Media containing the corresponding compounds were refreshed every 2 d. Total RNA was prepared using the RNeasyMini Kit (Qiagen), following the manufacturer’s instructions. To quantify the levels of *ALPL* and *COL1A1* mRNA, complementary DNA was prepared from total RNA using the Transcriptor Reverse Transcriptase and an anchored-oligo (dT)18 primer (Roche Applied Science). qPCR was performed using LightCycler FastStart DNA Master SYBR Green I and LightCycler detector (both from Roche Applied Science). Quantitative expression values were extrapolated from standard curves, and were normalized to the expression values of beta-2-microglobulin (*B2M*) and beta-glucuronidase (*GUSB*) which were used as endogenous controls. Specific oligonucleotide primers were*: COL1A1*, 5′-CGGGCCTCAAGGTATTGCT-3′ (forward primer, F) and 5′-GGGACCTTGTTTGCCAGGTT-3′ (reverse primer, R); *ALPL*, 5′-GACTAAGAAGCCCTTCACTGCCAT-3′ (F), 5′-GACTGCGCCTGGTAGTTGTT-3′ (R); *B2M*, 5′-CCAGCAGAGAATGGAAAGTC-3′ (F) and 5′-GATGCTGCTTACATGTCTCG-3′ (R); *GUSB*, 5′-AAACGATTGCAGGGTTTCAC -3′ (F), 5′-CTCTCGTCGGTGACTGTTCA-3′(R).

### Statistical analysis of data

Analysis of variance (ANOVA) of the results in each experiment for tested samples was performed with respect to LG-DMEM or differentiation LG-DMEM at each time and condition at significance level of *p < 0.05, **p < 0.01 and ***p < 0.001, and with respect to PA samples at each time and condition at significance levels of ^#^p < 0.05, ^##^p < 0.01 and ^###^p < 0.001.

## Supplementary information

Supplementary information

## Data Availability

Materials, data and associated protocols are available to readers without undue qualifications in material transfer agreements.
