# Supplementary material for: Glycerylphytate compounds with tunable ion affinity and osteogenic properties
Source: Sci Rep. 2019 Aug 7;9:11491. doi: 10.1038/s41598-019-48015-5 (PMC6685941; doi:10.1038/s41598-019-48015-5)
Supplement: Supplementary file 1 — Supplementary information [file 41598_2019_48015_MOESM1_ESM.docx]

Glycerylphytate compounds with tunable ion affinity and osteogenic properties

Ana Mora-Boza^1,2^‡, María Luisa López-Donaire^1^‡, Laura Saldaña^3,2^, Nuria Vilaboa^3^, Blanca Vázquez Lasa^1,2*^, Julio San Román^1,2^

^1^Institute of Polymer Science and Technology, ICTP-CSIC. C/ Juan de la Cierva 3, 28006 Madrid, Spain.

^2^CIBER-BBN. Health Institute Carlos III, C/ Monforte de Lemos 3-5, Pabellón 11, 28029 Madrid, Spain.

^3^Hospital Universitario La Paz-IdiPAZ, Paseo de La Castellana 261, 28046 Madrid, Spain

**SUPPORTING INFORMATION**

**
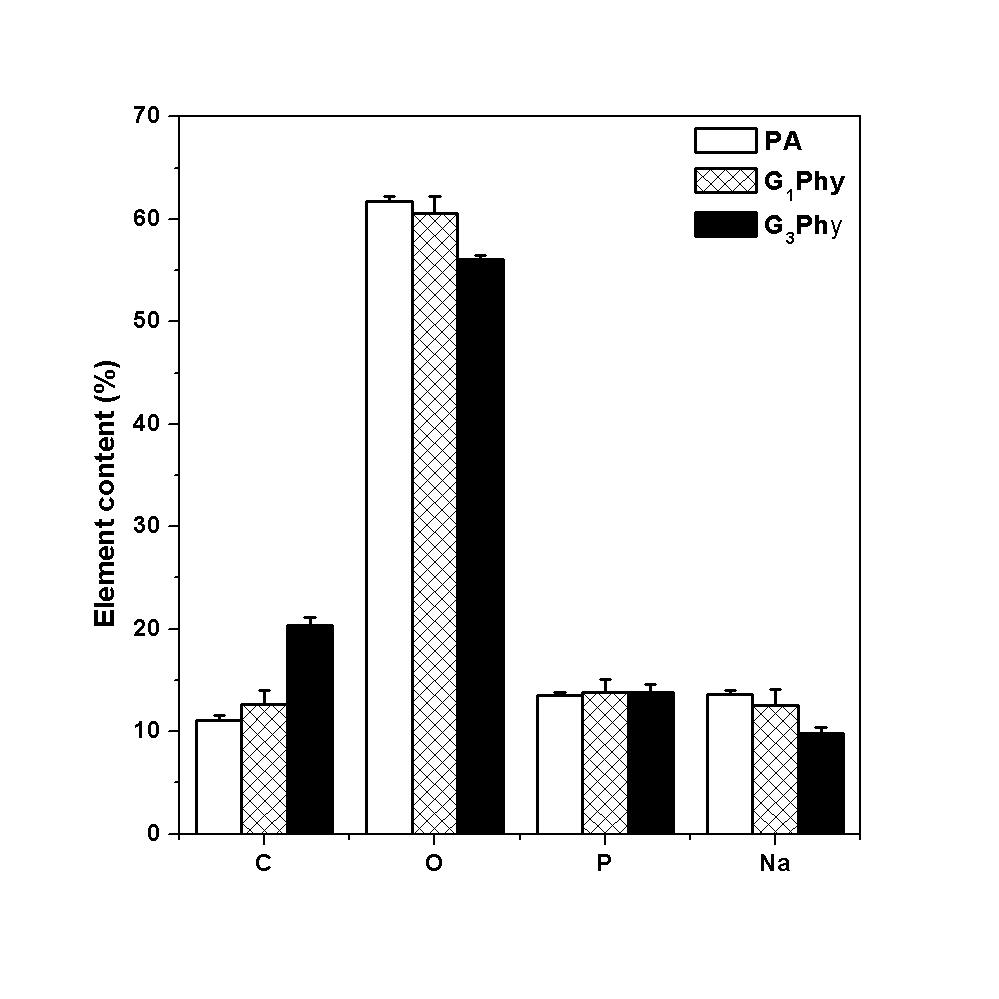
**

**Figure S1**. Elements content quantification of PA and GPhy derivatives by EDX

spectroscopy.

**Figure S2.** ^13^C NMR (a) and ^1^H NMR (b) spectra of G, PA and GPhy derivatives

recorded in D_2_O at 25ºC. For ^13^C experiments chemical shifts were referenced to

deuterated dioxane while the residual proton absorption of D_2_O (δ 4.79) was used as

external reference in ^1^H experiments.

**Table S1.** Thermal degradation results for G, PA and GPhy compounds under inert

atmosphere.

| Sample | T_max_ (ºC) | | | | Residue (%)  at 800 ºC |
| --- | --- | --- | --- | --- | --- |
|  | 1^st^ stage | 2^nd^ stage | 3^rd^ stage | 4^th^ stage |  |
| G | 228 |  |  |  | 0 |
| PA | 117 | 215 | 332 | 394 | 75 |
| G_1_Phy | 137 | 242 | 356 | - | 69 |
| G_3_Phy | 125 | 215 | 337 | - | 52 |
